# Two middle-aged cases of deep overbite without molar support treated by orthognathic surgery

**DOI:** 10.1016/j.ijscr.2019.09.027

**Published:** 2019-09-24

**Authors:** Kazuki Akizuki, Keiko Fujita, Hiroki Kobayashi, Yasuhito Tsuchida, Tsuyoshi Shimo

**Affiliations:** a Matsuda Orthopedic Memorial Hospital, Kita 18, Nishi 4 1-35 Kita-ku, Sapporo, Hokkaido, 001-0018, Japan; b Kobayashi Orthodontic Clinic, Kita 3, Nishi 3 1-41 Chuo-ku, Sapporo, Hokkaido, 060-0003, Japan; c Tsuchida Orthodontic Dental Office, Maeda 1 12-1-48 Teine-ku, Sapporo, Hokkaido, 006-0811, Japan; d Division of Reconstructive Surgery for Oral and Maxillofacial Region, Department of Human Biology and Pathophysiology, School of Dentistry, Health Sciences University of Hokkaido, 1757 Ishikari-Tobetsu, Hokkaido 061-0293, Japan

**Keywords:** Orthognathic surgery, Severe overbite, Middle-aged, Implant

## Abstract

**Introduction:**

Patients with severe overbite in middle age often undergo prosthetic treatment without a diagnosis of dentofacial deformity, but soft tissue trauma can occur in such patients due to the lack of inter-occlusal space. Comprehensive surgical orthodontic treatment and prosthetic treatment are indicated to correct the overbite and soft tissue injury for such patients.

**Presentation of case:**

We report the cases of two middle-aged patients with dentofacial deformity and severe overbite without molar support. In both cases, prosthetic treatment had been performed for many years without any improvement of the occlusion. Case 1: A 47-year-old Japanese man had a maxillary incisor protrusion and reduced lower anterior facial height profile with the left mandibular molars lost due to periodontal disease. After preoperative orthodontic treatment and occlusal elevation in the molar teeth using an implant prosthesis, a sagittal split ramus osteotomy (SSRO) was performed. Case 2: A 57-year-old Japanese woman had mandibular retrognathia with maxilla and mandibular-arch length discrepancy. The left mandibular molars needed to be extracted due to periodontal disease. After preoperative orthodontic treatment and reconstruction of the molar occlusion using an implant prosthesis, three-segment Le Fort I osteotomy and SSRO were performed.

**Discussion:**

Malocclusion with dentofacial deformity is a risk factor for severe deep overbite or other occlusion collapse.

**Conclusion:**

In middle-aged patients with deep overbite with missing molar teeth, we should consider both prosthodontic treatment and comprehensive dental therapy, including orthognathic surgery.

## Highlights

• A deep overbite is defined as a vertical overlap of the upper and lower incisors.
• Problems related to deep overbite can include soft tissue trauma.
• It is difficult to recover functions with ordinary prosthetic therapy alone in patients with severe deep overbite with dentofacial deformities.
• Comprehensive surgical orthodontic treatment is indicated for such patients.

## 1 Introduction

A deep overbite is defined as a vertical overlap of the upper and lower incisors that exceeds half of the lower incisal tooth height. Problems related to deep overbite can include soft tissue trauma, a lack of inter-occlusal space, and tooth wear. Restorative management of the deep overbite has been reported [1]. However, in patients with severe deep overbite with dentofacial deformities in which the positional relationship between the upper and lower jaws has vertical or horizontal incongruity, it is difficult to recover functions with ordinary prosthetic therapy alone. Such cases require occlusal reconstruction using a combination of different prosthetic treatments.

We report the cases of two middle-aged patients who had undergone prosthetic treatment for many years, resulting in dentofacial deformity that included severe overbite without molar support. In both cases, secure stable occlusion and satisfactory aesthetics were achieved through treatment with prosthodontic, comprehensive dental therapy and orthognathic surgery.

## 2 Methods

Two cases of middle-aged patients with dentofacial deformity and severe overbite without molar support. Written informed consent was obtained from the patients for publication of this case report and accompanying images. A copy of the written consent is available for review by the Editor-in-Chief of this journal on request. This research work has been reported in line with the PROCESS criteria [2].

## 3 Presentation of cases

**Fig. 1:**
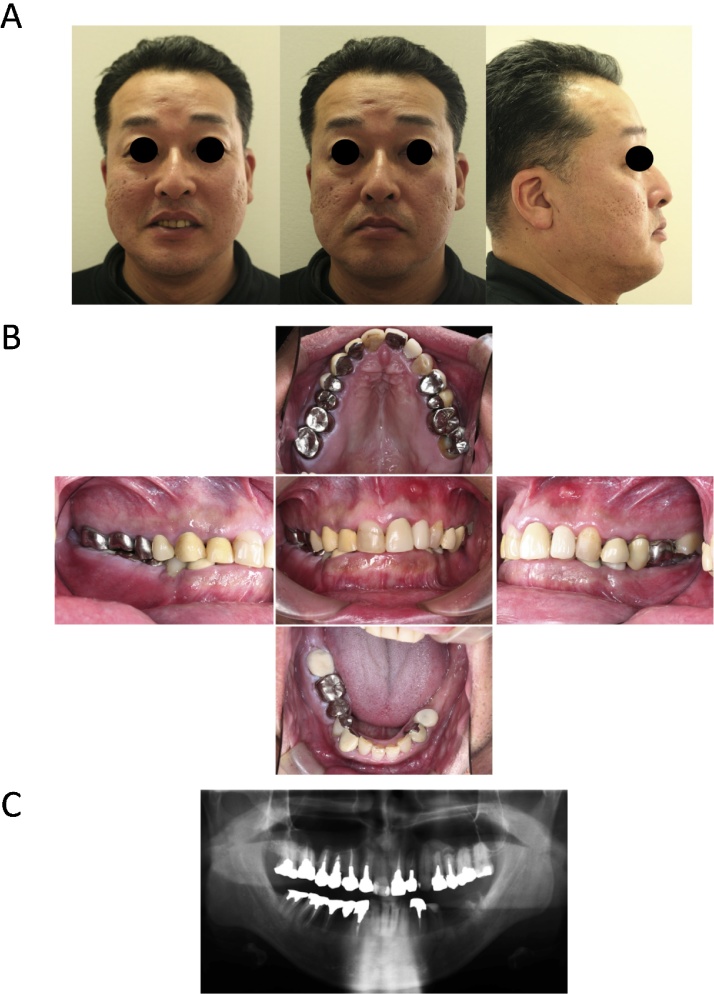
Extraoral and intraoral photographs and panoramic X-ray at pretreatment in Case 1.

Case 1: A 47-year-old male came to our hospital with a main complaint of occlusal imperfection. He had undergone continuing prosthodontic treatment at a primary care dental clinic, but the occlusal trauma on the lip side of the anterior lower gingiva had progressed over time due to the loss of the lower left molars and to periodontal disease. He had a maxillary incisor protrusion and reduced lower anterior facial height profile (Fig. 1A). Intraoral examination revealed a class III malocclusion with an excessive overbite (10 mm) and overjet (5 mm) and a lack of inter-occlusal space (Fig. 1B and C). The patient was diagnosed as having a deep bite malocclusion with maxilla and mandibular-arch length discrepancy. After the left mandibular molars with periodontitis were extracted, the bilateral molars were reconstructed by placing implants and prosthesis, resulting in a temporary elevation of their bite. The patient declined operation on the upper jaw osteotomy due to concern over the potential impact on aesthetics, and therefore only the lower jaw advancement was planned.

**Fig. 2:**
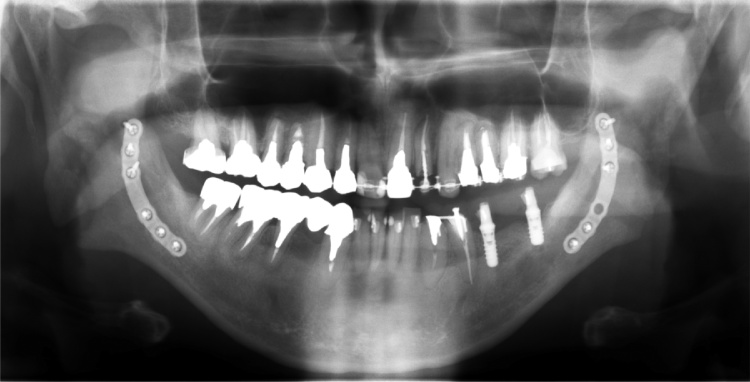
Panoramic X-ray at 3 months after the surgery in Case 1.

After pre-surgical orthodontic treatment and prosthesis treatment, a mandibular bilateral sagittal split ramus osteotomy (SSRO) was performed under general anesthesia. The bilateral mandible distal bone fragment was advanced 6 mm and the fixation was performed using a 6-hole titanium plate (AO Matrix Mandible plate, 1.25 mm; DePuy Synthes, Zuchwil, Switzerland). The inter-maxillary fixation was started on the day after the operation by using orthodontic elastics and continued for 3 months. Piperacillin sodium was administered by intravenous infusion at 1 g × 2/day for 3 days. A liquid diet was started on the first day after the operation, and beginning on the fifth postoperative day the meal grade was gradually increased to normal. After discharge, follow-up was performed on an outpatient basis every few weeks until 3 months after surgery, and it was confirmed that the postoperative course was good. The results of cephalometric analysis at 1 month after operation were as follows: overbite, 2 mm; overjet, 2.5 mm; sella-nasion to point A angle (SNA), 85.9° (mean 81.8°, standard deviation (SD) 0.8); sella-nasion to point angle B (SNB), 81.6° (mean 78.1°, SD 0.7); A point–nasion–B point angle (ANB), 4.3° (mean 3.8°, SD 0.3); mandibular plane (MP) to sella-nasion plane angle (MP-SN), 41.32° (mean 37.6°, SD 0.5); and gonial angle, ramus plane to MP angle, 126.2° (mean 125.2°, SD 0.13). Fig. 2 shows the panoramic X-ray view at 3 months after the surgical treatment. The occlusal stabilization was obtained followed by the completion of the postsurgical orthodontic and bilateral molar occlusal reconstruction.

**Fig. 3:**
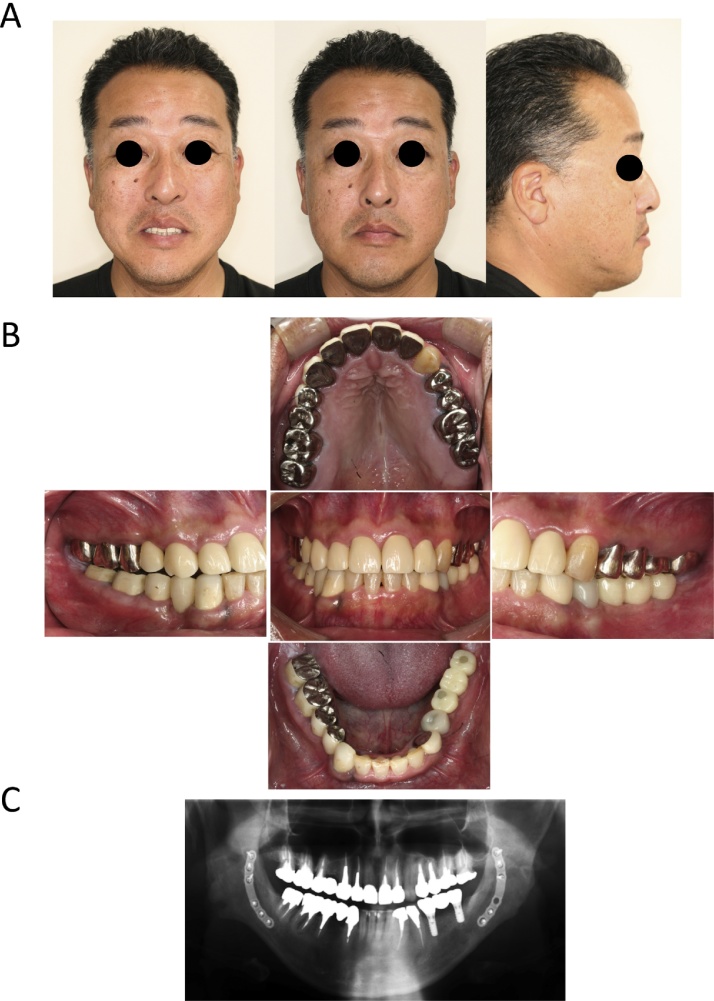
Extraoral and intraoral photographs and panoramic X-ray at debonding in Case 1.

Fig. 3A–C show the extraoral and intraoral views and panoramic X-ray view at 3 years after the surgical treatment. The patient reported satisfaction at being able to use his front teeth when eating, and said that others had observed that he was laughing more frequently. The occlusion is currently stable and the patient reports that it is no longer hindering his daily life. Follow-up has been continued on an outpatient basis to 5 years after surgery.

**Fig. 4:**
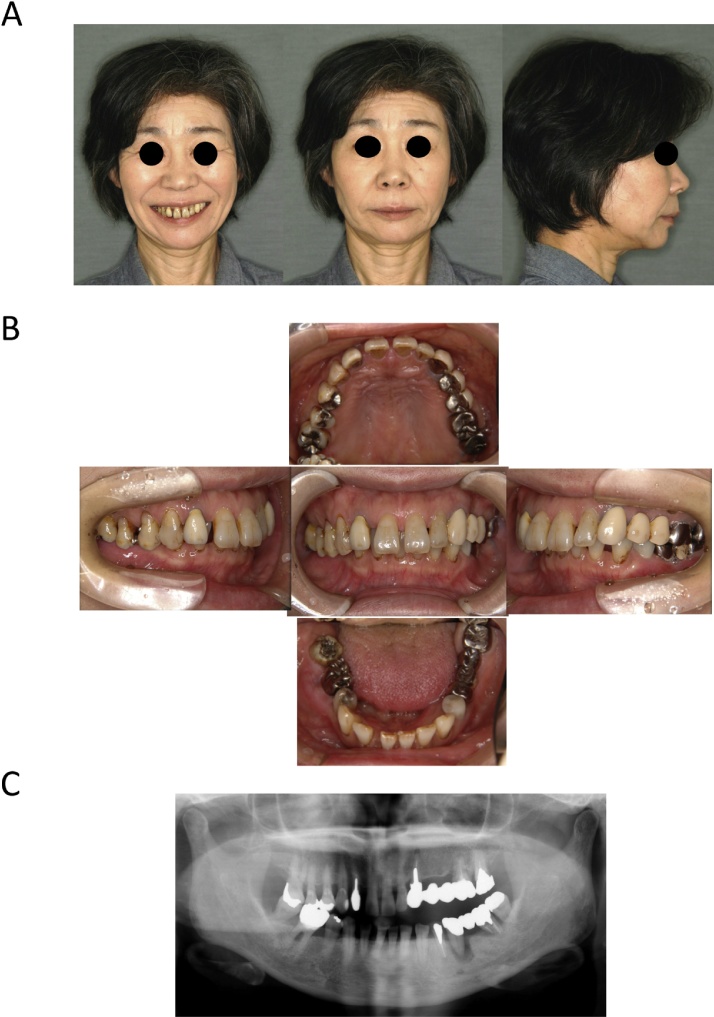
Extraoral and intraoral photographs and panoramic X-ray at pretreatment in Case 2.

Case 2: A 58-year-old female had consciously recognized an occlusion abnormality since she had been a high school student, but she had previously undergone only prosthodontic treatment at a primary care dental clinic. She visited our hospital when the occlusal imperfection and occlusal trauma of the palatal side of the gingiva at the anterior and right molars worsened. She had a mandibular retrognathia and facial asymmetry (Fig. 4A). Intraoral examination revealed a class II malocclusion with an excessive overbite (7 mm) and overjet (6 mm). She lacked an inter-occlusal relation at the left incisor to the right molars, which induced occlusal trauma at the palatal gingiva at that site because the maxilla arch length was wider than the mandible arch length (Fig. 4B). The left lower molar teeth had a bridge attached, but there was mobility due to periodontal disease (Fig. 4C). The patient was diagnosed as having an angle class II malocclusion with a skeletal class II jaw base relationship, maxillary cant, and a dental-arch width discrepancy. After pre-surgical orthodontic treatment and the reconstruction of the molar occlusion using an implant prosthesis, a maxillary 3 segmental Le Fort I osteotomy for dental-arch width narrowing and cant correction and a mandibular bilateral SSRO were performed. The maxilla was divided into three blocks of 654┛ (right), 321┻123 (anterior), and ┗67 (left) region after downfracture of the Le Fort I osteotomy. The repositioned maxilla was impacted 5 mm at the 654 ┛ region and narrowed with a width of 8 mm at the molar part. The maxilla was fixed using a 1.7 mm-thick universal Le Fort I titanium plate and L-shaped titanium plate (Stryker, Kalamazoo, MI) in two locations, anterior and posterior. The mandibular bone fixation was performed by using 6-hole (right) and 4-hole (left) titanium plates (AO Matrix Mandible plate, 1.25 mm; DePuy Synthes). The inter-maxillary fixation was started on the day after the operation by using orthodontic elastics and continued for 3 months. Piperacillin sodium was administered by intravenous infusion at 1 g × 2/day for 3 days. A liquid diet was started on the first day after the operation, and beginning on the fifth postoperative day the meal grade was gradually increased to normal. The outpatient follow-up was performed every few weeks until 3 months after surgery.

**Fig. 5:**
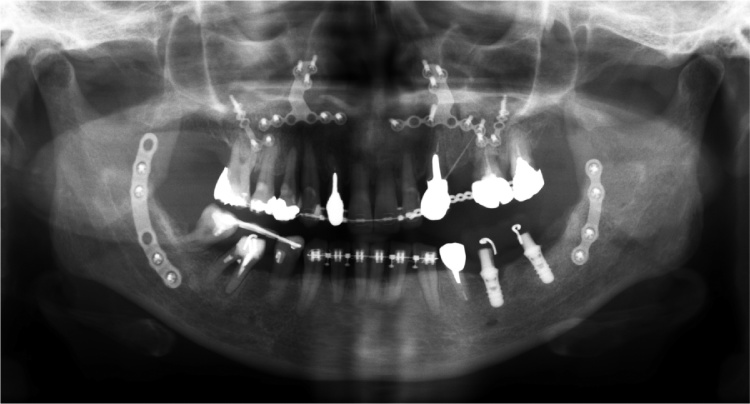
Panoramic X-ray at 6 months after the surgery in Case 2.

**Fig. 6:**
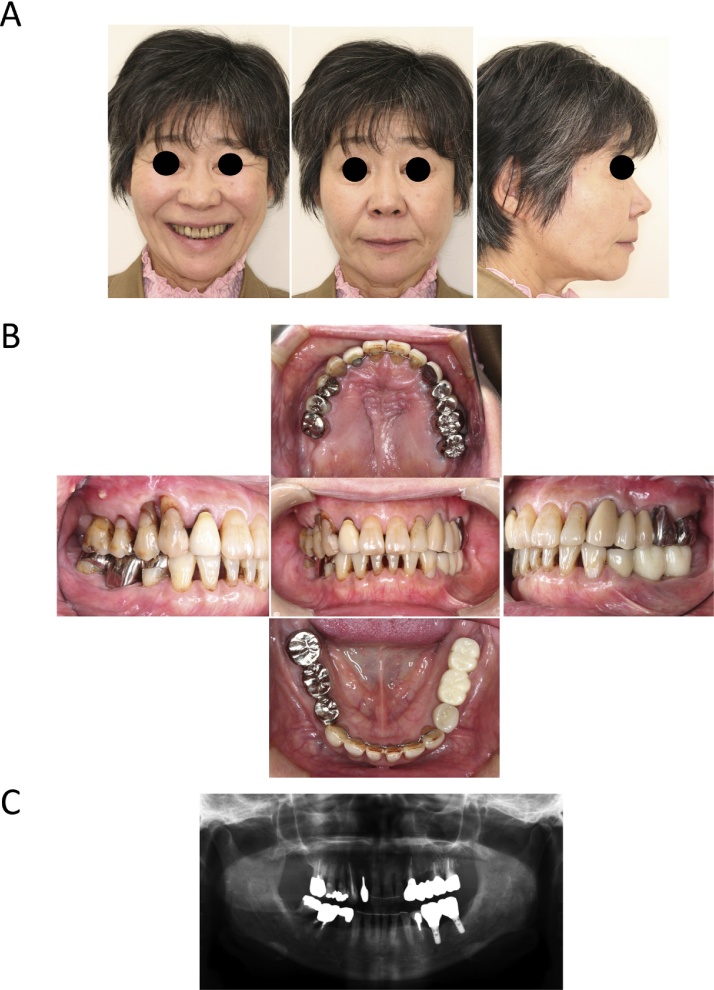
Extraoral and intraoral photographs and panoramic X-ray at debonding in Case 2.

Fig. 5 shows the panoramic X-ray view at 6 months after the surgical treatment. Occlusal stabilization was obtained followed by the completion of the postsurgical orthodontic and bilateral molar occlusal reconstruction. Fig. 6A–C show the extraoral and intraoral views and the panoramic X-ray view at 3 years after the surgical treatment. At 3 years after the initial treatment, the patient showed a skeletal class I jaw relationship and the representative initial cephalometric values were improved as follows: A–B plane angle, point A-point B to nasion-pogonion angle (mean −4.8°, SD, standard deviation 3.5) −11.3° → −4.7°; SNA, sella-nasion (SN) to point A angle (mean 82.3°, SD 3.5) 85.0° → 82.3°; SNB, SN to point B angle (mean 78.9°, SD 3.5) 77.3° → 78.0°; ANB, A point, nasion, B point angle (mean 3.4°, SD 1.8), 7.8° → 4.3°; MP-SN, mandibular plane (MP) to SN plane angle (mean 40.2°, SD 4.6) 35.6° → 39.1°; MP-FH, MP to frankfort (FH) plane angle (mean 28.8°, SD 5.2) 24.6° → 28.0°; Gonial angle, ramus plane to MP angle (mean 131.0°, SD 5.6) 120.2° → 127.7°. The patient reported that she no longer experienced damage to the palatal mucosa from her lower anterior teeth when eating, and meals had become fun. Above all, she was pleased that she no longer hid her mouth with her hand during conversation, that she felt more motivated to dress up, and that she had more opportunities to go out. Her occlusion is currently stable and she is satisfied with her aesthetics at the follow-up 5 years after surgery.

## 4 Discussion

Severe deep overbite is one of the most common malocclusions in orthodontic practice [3]. Indeed, when defined as overbite ≥5 mm, the condition is found in nearly 20% of children and 13% of adults, and accounts for about 95.2% of the vertical occlusal problems [4]. It is widely accepted that orthodontic treatment of severe deep overbite is both easier to accomplish and more stable when performed on growing patients than when performed on adults. For this reason, adults often avoid orthodontic treatment and settle for simple correction of excessive overbite. For patients who do not complain about facial aesthetics, camouflage therapy—in which skeletal problems are masked by rearranging the dentoalveolar structure—can be offered [5]. Deep overbite can be classified as dentoalveolar deep overbite and skeletal deep overbite, true deep overbite and pseudo deep overbite or incomplete deep overbite and complete deep overbite [6]. For skeletal deep overbite, better results can be achieved with a combination of surgical and orthodontic treatment than by orthodontics alone [7,8]. However, the majority of middle-aged patients with deep overbite are asymptomatic, and where the appearance is aesthetically acceptable patients are unlikely to seek treatment [9]. The problems associated with middle-aged patients with severe deep overbite can undoubtedly present clinical challenges.

In middle-aged patients with severe deep overbite, treatment planning consisting of mere cephalometric evaluation and clinical observations is not always sufficient, and external factors such as environment, family, friends, and business life must be considered when planning treatment. As the number of patients with systemic illness in middle age has increased in recent years, patient tolerance of treatment should also be considered when planning treatment, along with the expected longevity and the maintenance burden of various options [10]. In such cases a long-term treatment plan with a great deal of patient involvement is often required.

The treatment should be aimed at improving occlusal stability, function, and aesthetics, but in middle-age patients with overbite, soft tissue trauma reduction and periodontal health improvement should also be considered. In our Case 1, elevation of the bilateral molar bite provided good support for the lower lip, while in Case 2, maxillary segmental narrowing provided a satisfactory width relationship with the mandible. Deep overbite does not involve tooth-to-teeth contact between the incisors, but contact between the mandibular incisors and palatal mucosa can cause mucosal pain and swelling as well as dysfunction. Progression from asymptomatic deep overbite to symptomatic traumatic overbite in middle-aged and elderly patients has been reported to be due to several factors [8]. Deep overbite tends to induce soft tissue trauma from the opposite incisions, and the resulting discomfort hinders oral hygiene efforts and increases the risk of periodontal disease [1].

In Case 1, the lingual inclination of the lower incisors was suggested to be induced by the presence of a strong lower lip and the loss of support for the left lower molars; the margin of the lower incisors may then have led to biting into the palatal mucosa [11]. In Case 2, dental bone loss and excessive closure in the anterior direction of the mandible were induced with the loss of posterior occluding units due to periodontal disease, and soft tissue trauma may have occurred due to the mandibular anterior teeth margin [12]. Also, the larger diameter of the upper jaw compared to the lower jaw caused the lower right molars to come into contact with the palatal mucosa. Both cases showed no improvement over time with prosthodontic treatment, which should aim to optimize periodontal health and improve occlusal stability, function, and aesthetics. In both our cases the treatment was divided into two stages: orthognathic surgery for the restoration of occlusal stability and function, and crown restoration treatment accompanied by improvement of the occlusal diameter. Where there is a significant underlying skeletal discrepancy, orthodontics alone is unlikely to produce a stable inter-incisal relationship. In such cases, multidisciplinary management may be required. The preoperative and postoperative orthodontic treatment of patients with many teeth missing due to dental caries and periodontal disease is different from the treatment of patients without endodontic and periodontal diseases. In such cases, minimal tooth movement focused on occlusal recovery is performed at the time of preoperative orthodontic treatment, so as not to cause the arrangement of the remaining teeth to deteriorate.

In both Cases 1 and 2, because the left lower mandibular molar was missing during the preoperative orthodontic treatment, we placed an implant in order to realize intermaxillary fixation during and after the orthognathic surgery and thereby stabilize and improve the occlusal diameter. In cases involving mandibular orthognathic surgery for patients with mandibular protrusion who have multiple tooth defects, a stable jaw position has been obtained by using temporary dentures [13]. In cases in which temporary dentures are used to stabilize the jaw position, post-operative intermaxillary fixation is performed for a longer period than usual to promote healing and manage pain [13]. However, in the present case, the jaw position was secured by occlusal elevation using implant placement, which could be performed in the usual intermaxillary fixation period.

The number of cases in which implants are selected as a prosthetic treatment for missing teeth will increase as implant treatment becomes more widespread. On the other hand, in implant treatment for patients with dentofacial deformities, it is necessary to consider the order and timing of the treatment on a case-by-case basis. The malocclusion and jaw function abnormalities associated with dentofacial deformities can be considered to be risk factors related to occlusion and to the collapse of occlusion along with factors such as dental caries/periodontal disease and aging [14]. Therefore, in cases with multiple tooth loss and dentofacial deformity, a comprehensive dental treatment incorporating orthognathic surgery should be considered as an option, not only for prosthodontic treatment of the defect but also to obtain a stable occlusion in the long run. Therefore, cooperation is important not only between orthodontic dentists and oral surgeons, but also among prosthodontists such as general dentists, conservative doctors, and preventive dentists.

## Funding

None to declare.

## Ethical approval

The study was approved by the Matsuda Orthopedic Memorial Hospital.

## Consent

Consent was taken from the patient involved. The patient in question has been de-identified. A copy of the written consent is available upon request.

## Author’s contribution

Kazuki Akizuki: Main surgeon involved in care of patient and final editor of manuscript.

Keiko Fujita: Collection of data and editing of manuscript.

Hiroki Kobayashi: Orthodontic treatment in Case 2.

Yasuhito Tsuchida: Orthodontic treatment in Case 1.

Tsuyoshi Shimo: Conceptualising and writing of the paper.

## Registration of research studies

researchregistry4951.

## Guarantor

Kazuki Akizuki.

Tsuyoshi Shimo.

## Provenance and peer review

Not commissioned, externally peer-reviewed.

## Declaration of Competing Interest

None to declare.
